# Inequities in the Geographic Accessibility of COVID-19 Biomedical Therapeutic Trials in the United States

**DOI:** 10.1007/s11606-021-07081-0

**Published:** 2021-09-03

**Authors:** Rohan Khazanchi, Samuel D. Powers, Elizabeth T. Rogawski McQuade, Kathleen A. McManus

**Affiliations:** 1 College of Medicine, University of Nebraska Medical Center, Omaha, NE USA; 2 University of Minnesota School of Public Health, Minneapolis, MN USA; 3 Division of Infectious Diseases and International Health, Department of Internal Medicine, University of Virginia, PO Box 801379, Charlottesville, VA 22908 USA; 4 Department of Public Health Sciences, University of Virginia, Charlottesville, VA USA; 5 Global Infectious Disease Institute, University of Virginia, Charlottesville, VA USA

**Keywords:** Coronavirus disease 2019 (COVID-19), Clinical trials, Rural health, Health disparities, Access to care

## Introduction

Coronavirus disease 2019 (COVID-19) has disproportionately impacted marginalized communities across the United States (US).^1^ However, racial/ethnic minority and elderly populations experiencing the highest COVID-19 incidence, hospitalization, and mortality rates have not been equitably enrolled in clinical trials investigating potential COVID-19 therapeutics.^2,3^

We descriptively evaluated the geographic proximity of demographic subpopulations to COVID-19 biomedical therapeutic trial sites. We hypothesized that trial sites would be more accessible to urban populations and subgroups who more often live in urban areas (racial/ethnic minority and younger populations).

## Methods

For this cross-sectional analysis, we queried ClinicalTrials.gov for trials with keywords “*coronavirus disease 2019*,” “*COVID-19*,” and “*SARS-CoV-2*” and start dates between January 20th and September 20th, 2020. To identify biomedical therapeutic trials, two authors excluded observational, suspended, terminated, withdrawn, and non-therapeutic trials.

We geocoded trial site addresses using Google Places API. We calculated drive times from the center of population for each census tract to the ten geographically closest sites and selected the site with the shortest time. We stratified rural and urban tracts using 2010 USDA ERS Rural-Urban Commuting Area codes. We calculated the proportion of each demographic subgroup residing within *x* minutes of the nearest trial site by weighting each tract by population demographics (age, race, ethnicity) from the 2015–2019 US Census American Community Survey (ACS). We calculated median drive times with 95% confidence intervals by bootstrap.

We performed statistical analyses using RStudio v1.3.1073 (R Foundation for Statistical Computing), and plotted maps using ArcMap v10.7.1. The University of Virginia Institutional Review Board deemed this study exempt.

## Results

We identified 310 biomedical therapeutic trials with 2095 trial sites, including 246 (79.4%) randomized trials. Median trial enrollment was 117 (IQR 335). Most trials included all genders (307 [99.0%]) and adults older than 18 years (285 [91.9%]). One hundred seventy-two (55.5%) were single-center studies (range 1–117 sites). The most studied interventions included convalescent plasma (37 [11.9%]), hydroxychloroquine (25 [8.1%]), and remdesivir (11 [3.5%]).

**Figure 1:**
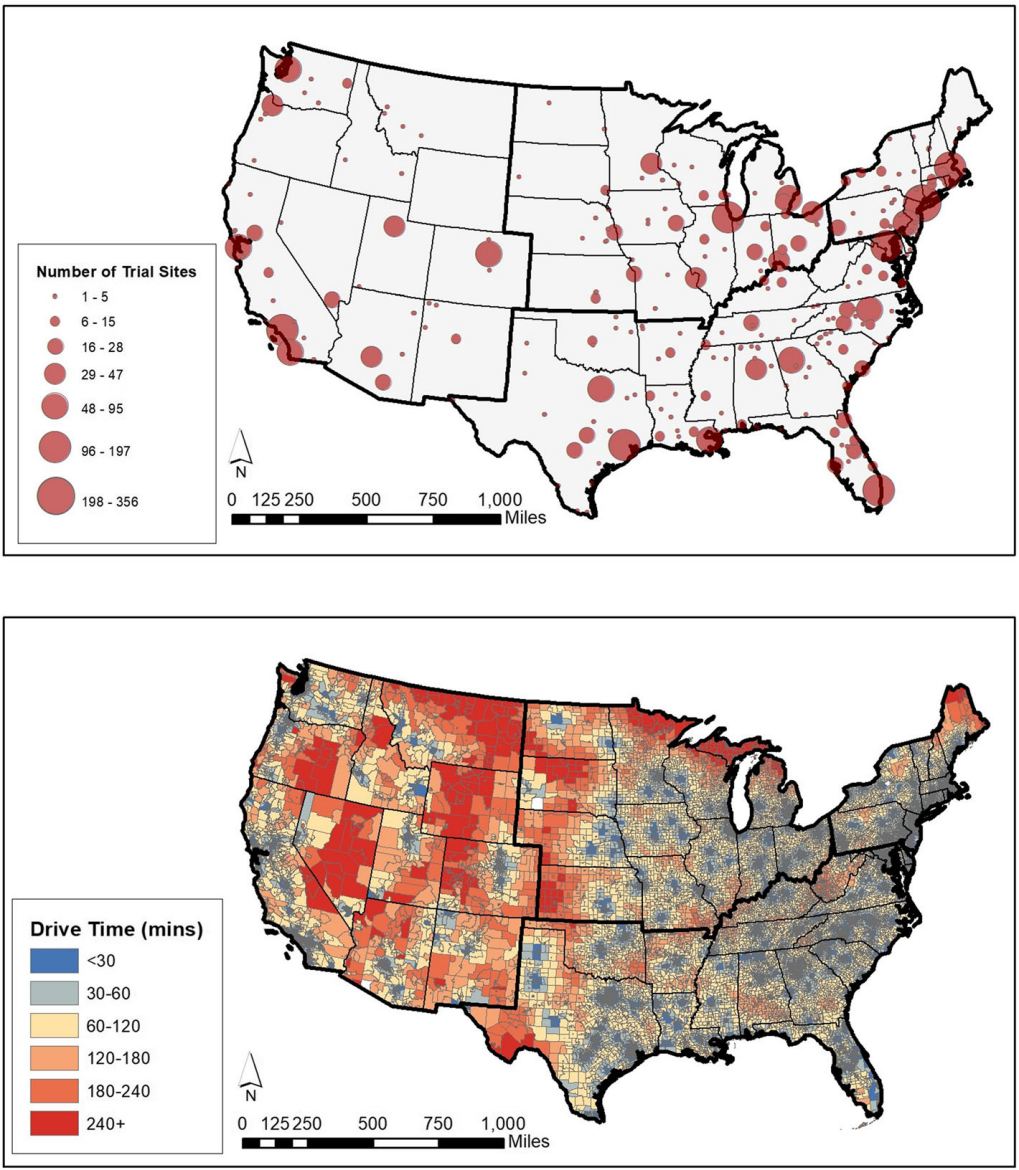
(A) Geographic distribution and density of COVID-19 biomedical therapeutic trials across the contiguous United States. To display the number of trial sites within an area of geographic proximity, all trial sites were plotted and those within 25 miles of each other were aggregated into polygons. Circles representing the number of aggregated trial sites were plotted at the centroid of each polygon. (B) One-way drive time from census tract centers of population to the nearest COVID-19 biomedical therapeutic trial.

Trial sites were clustered near metropolitan centers (Figure 1A), with corresponding shorter drive times near urban areas (Figure 1B). Overall, 31.3% of the US population and 76.0% of the rural population lived > 60 min from the nearest trial site. 33.7% of elderly (age 65+), 56.3% of American Indian/Alaskan Native (AIAN), 32.8% of White, 18.5% of Hispanic, and 10.7% of Black people lived > 60 min from the nearest site.

**Figure 2:**
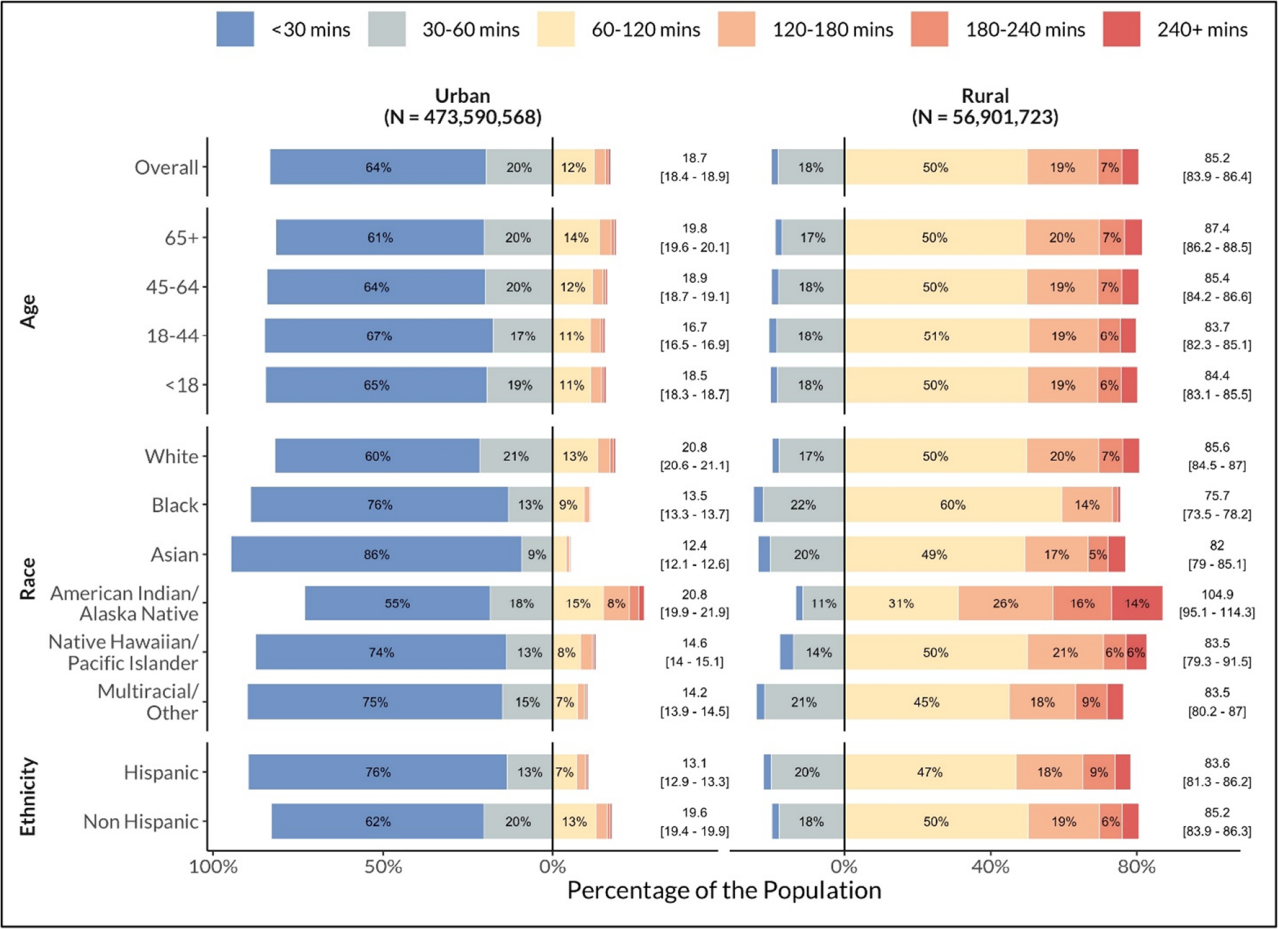
One-way drive times to the nearest COVID-19 biomedical therapeutic trial site for demographic subgroups, stratified by rurality.﻿ Bar graphs display the percentage of the population with less than (i.e., left of 0% on the *x*-axis) or greater than (i.e., right of 0% on the *x*-axis) a 60-min drive time to the nearest COVID-19 biomedical therapeutic trial site. For each sociodemographic subgroup, the median and 95% confidence interval are displayed to the right of the bar.

Rural census tracts (median 85.2 mins [95% CI: 83.9–86.4]) had significantly longer drive times than urban tracts (18.7 [18.4–18.9]) for all demographic groups (Figure 2). After stratifying by rurality, only median drive times for AIAN people were still significantly longer than drive times for the overall population in both urban (AIAN: 20.8 [19.9–21.9]; overall: 18.7 [18.4–18.9]) and rural (104.9 [95.1–114.3]; 85.2 [83.9–87.8]) tracts.

## Discussion

Similar to the geographic inaccessibility of clinical trials for other diseases,^4^ the opportunity to enroll in biomedical therapeutic trials throughout the first 8 months of the COVID-19 pandemic was not equitably available across the US. Nearly one-third of the overall US population, over one-half of AIAN people, and over three-fourth of the rural population lived more than an hour from the nearest trial site. Rural-urban differences in trial distribution explain longer overall drive times for White and elderly populations, since these groups disproportionately resided in rural census tracts. However, the AIAN population faced longer drive times even when accounting for rurality, suggesting they are uniquely geographically isolated from novel therapeutics.

Non-Hispanic and White individuals were well-represented in COVID-19 trials despite rural trial inaccessibility and lower hospitalization rates.^2^ Furthermore, the underrepresentation of Black and Hispanic populations in COVID-19 therapeutic trials is especially striking given their relative geographic proximity to trial sites and disproportionate hospitalization rates, both of which suggest greater opportunity for recruitment.^2^ Factors unexplored herein—including racism, mistrust, language barriers, and the persistent segregation of well-resourced hospitals—should be investigated further as potential mediators of decreased trial enrollment.^5^

Our study has limitations, including that our use of tract centers of population assumes demographic groups are not clustered within tracts. We also did not account for vehicle access or reliance on public transportation. Thus, our tract-level analyses may misestimate travel times for vehicle-less and demographically segregated urban populations.

Beyond the COVID-19 era, innovations like decentralized, Internet-based clinical trials may help mitigate geographic inequities.^6^ However, it remains clear that geographic accessibility alone may not improve racial/ethnic representation in the absence of additional structural interventions.

## Data Availability

The data analyzed for this study are publicly available from http://clinicaltrials.gov/.

## References

[1] Khazanchi R, Beiter ER, Gondi S, Beckman AL, Bilinski A, Ganguli I (2020). County-Level Association of Social Vulnerability with COVID-19 Cases and Deaths in the USA. J Gen Intern Med..

[2] Chastain DB, Osae SP, Henao-Martínez AF, Franco-Paredes C, Chastain JS, Young HN (2020). Racial Disproportionality in Covid Clinical Trials. N Engl J Med..

[3] Helfand BKI, Webb M, Gartaganis SL, Fuller L, Kwon C-S, Inouye SK (2020). The Exclusion of Older Persons From Vaccine and Treatment Trials for Coronavirus Disease 2019—Missing the Target. JAMA Intern Med..

[4] Galsky MD, Stensland KD, McBride RB (2015). Geographic Accessibility to Clinical Trials for Advanced Cancer in the United States. JAMA Intern Med..

[5] Warren RC, Forrow L, Hodge DA, Truog RD (2020). Trustworthiness before Trust — Covid-19 Vaccine Trials and the Black Community. N Engl J Med..

[6] Gaba P, Bhatt DL (2020). The COVID-19 pandemic: a catalyst to improve clinical trials. Nat Rev Cardiol..

